# Nutritional Interventions for Early Dementia

**DOI:** 10.1007/s12603-021-1616-4

**Published:** 2021-03-15

**Authors:** Matteo Cesari, D. Azzolino, B. Arosio, M. Canevelli

**Affiliations:** 1 Department of Clinical Sciences and Community Health, University of Milan, Milan, Italy; 2 Geriatric Unit, IRCCS Istituti Clinici Scientifici Maugeri, Via Camaldoli 64, 20138, Milan, Italy; 3 Geriatric Unit, Fondazione IRCCS Ca' Granda Ospedale Maggiore Policlinico, Milan, Italy; 4 Department of Human Neuroscience, Sapienza University, Rome, Italy; 5 National Center for Disease Prevention and Health Promotion, Italian National Institute of Health, Rome, Italy

**Keywords:** Aging, cognition, supplementation, nutrition, geriatrics, neurology

## Abstract

Nutrition plays a critical role in the definition of the individual's wellbeing. Nutritional interventions have been repeatedly advocated as of potential interest for preventing or delaying the cognitive decline, also in the context of neurodegenerative conditions. The idea of targeting the initial phases of dementia, when the process is theoretically still amenable of correction, via lifestyle modifications (including healthy diet or supplementation of specific micro-/macro-nutrients) is extremely appealing. In this perspective paper, we describe the most recent evidence on the topic and discuss how the nutritional assessment should be nested within a comprehensive approach to the aging person with initial signs of dementia for promoting his/her optimal management.

## Introduction

Nutrition and dementia have been graphically presented as closely connected in a vicious cycle in recent guidelines published by the European Society for Clinical Nutrition and Metabolism (ESPEN) (1). Cognitive impairment may affect nutrition leading to a decrease of nutrients intake. The resulting weight loss and nutritional deficits may thus worsen the cognitive function of the individual, and concur at developing ancillary geriatric conditions (e.g., frailty, sarcopenia) (2, 3, 4). In other words, whereas adequate nutrition represents a critical component of healthy ageing in every individual, this becomes particularly true in the frailest persons as those living with dementia (5, 6). In fact, the neurological condition might implicitly expose the person to an increased vulnerability to stressors. Therefore, every reversible risk factor (such as decreased dietary intake, poor quality of foods, social isolation) should be carefully evaluated and corrected for preventing the onset of the disabling process (7).

A person with early dementia, usually characterized as having a Clinical Dementia Rating score of 1, is characterized by abnormalities at cognitive tests and has started experiencing difficulties in the conduction of his/her daily activities. The functional abnormalities can affect different domains (e.g., memory, orientation, judgment and problem solving, social activities, hobbies, personal care, affects) and in different ways. The result is that the phenotypic expression of a person with early dementia is usually extremely heterogeneous under biological, clinical, and social perspectives (8). Interestingly, some common issues found in persons with early dementia may play a relevant role in the alteration of nutritional patterns. For example, changes in olfactory and taste discrimination/identification and food preferences, attention deficits, executive function deficits, impaired decision-making ability, and behavioral expressions (e.g., apathy, depression) may represent early manifestations in dementia altering the capacity of the individual to properly acquire the quantity and quality of needed nutrients (1, 9).

Nevertheless, despite the initial modifications of behaviors, persons with early dementia may not present major differences in nutritional status compared to healthy individuals. A recent study by Olde Rikkert and colleagues (10) compared a group of patients with mild Alzheimer's disease (AD) versus healthy controls for multiple micronutrients, anthropometric measures, and malnutrition assessment tools. Only few, relatively small, and far to be clinically relevant differences were found. A recent systematic review consistently showed that energy and protein intake in patients with AD is not substantially different from cognitively normal controls (11).

If the micro- and macronutrient intakes are not substantially different at the early stages of the dementia process, it might then be assumed that the general recommendations available for older persons directly apply to this group of patients. Indeed, the clinical consequences of an early stage disease may not yet produce evidence of major nutritional changes. Considering that older persons often presents multimorbidity (12, 13), it might be important to weight the burden of (early) dementia in the overarching clinical complexity of the individual. It becomes crucial to evaluate whether the cognitive dysfunction may simply be one of the many issues, without necessarily play a significant role in the definition of the patient's priorities.

In other words, whereas the dementia condition has not affected the nutritional status of the individual, the traditional recommendations available for promoting healthy aging in older persons can apply. Therefore, setting the optimal intake of dietary proteins to 1.0–1.2 g/kg of body weight per day may become applicable independently of the presence of early dementia (14, 15). In case of concurrent acute illnesses, early dementia may play the role of an irrelevant comorbidity under the nutritional perspective, and the protein intake might simply be increased to 1.2–1.5 g/kg of body weight per day as in persons without the neurodegenerative condition. Consistently, the evidence suggesting to 1) spread the dietary protein intake over different meals throughout the day (2), and 2) practice regular physical activity (14, 15) remains valid. In a person at the beginning of a clinical course characterized by increasing loss of autonomy, the maintenance of physical function (largely based on muscle health) becomes pivotal (16). For this reason, behaviors enhancing the protein synthesis and muscle preservation are actions of special relevance in the context of dementia (17). Another important aspect to consider in older persons with early dementia is the possible deficiency of vitamin D, especially in those individuals who may have reduced their outside activities (18, 19). It is today demonstrated that the vitamin D acts as a pre-hormone; its receptor is not only located in bones but also in muscles (20). It is thus clear why the supplementation should be considered when low concentrations are reported. Furthermore, it is noteworthy how low concentrations of vitamin D have been associated with poor cognitive functioning and indicated as potentially involved in the incidence/worsening of dementia (21).

When discussing about nutritional needs in older persons and looking for possible solutions, the dramatically high prevalence of malnutrition should not be overlooked (22). Kaiser and colleagues (23) reported that more than one third of community-dwelling older persons are at risk of malnutrition or already malnourished. In this setting, there are likely many individuals with the first signs of dementia (24). The figures are even worse when observing other settings (e.g., hospital, rehabilitation) where persons with early dementia might be referred. Again, the early stage of the neurodegenerative disease might be a simple comorbidity in a subject with dysfunctions unrelated to the cognitive domain. The critical point is to detect the nutritional need, something that too frequently is neglected in the standard clinical care (22, 25).

In this context, we need to consider how malnutrition is the result a multidimensional mechanism (26). Social changes (e.g., poverty, living alone, eating alone), physiological modifications (e.g., sensory impairment, poor oral health, gastrointestinal disorders, lack of appetite), and psychological problems (e.g., depression, lower motivation) may heterogeneously combine, causing abnormalities in the eating process and consequent modification of food quality and quantity (27). It is also important to recall the so-called anorexia of aging phenomenon acting as underlying phenomenon predisposing to the onset of undernutrition (28, 29).

As above-mentioned, multimorbidity is a common feature of frail older persons (30, 31), and different clinical conditions might contribute at determining malnutrition in dementia through different mechanisms (32). For example, diseases can be responsible of poor protein and energy intake by increasing metabolism, generating anorexia, causing swallowing difficulties, and/or enhancing the age-related malabsorption of nutrients. In other words, every manifestation of malnutrition should be examined and evaluated on a case-by-case.

The complexity of nutrition in the context of dementia is exemplified in a recent document by Alzheimer's Disease International where the connection between poor oral health and cognitive impairment is shown (33). In particular, many nutrition-related factors (e.g., chewing capacity, infections, quantitative and qualitative adaptations of diet) are indicated as possible determinants of the cognitive decline. If the scenario becomes clearly difficult to disentangle because multiple systems (as well as social factors) are involved, many opportunities for preventing/reversing the vicious cycle of malnutrition arise. This means that, as also stated by the World Health Organization (WHO) in the World Report on Ageing and Health, “the management of malnutrition in older age needs to be multidimensional” (34).

Merging the need to be comprehensive and personalize interventions, the nutritional management of older persons with early dementia may thus be focused at those clinical features characteristic of the condition of interest (35). If the person with early dementia may not substantially different from a healthy individual under nutritional aspects, it is however true that some of his/her symptoms have to be considered in the design of a person-tailored preventive or therapeutic program on (the risk of) malnutrition. As such, attention deficits, executive function deficits, and impaired decision-making ability may be present and should be targeted by specific interventions when needed (1). For example, attention deficits may cause difficulties in shopping, preparing meals, and/or eating regularly. The solution might be represented by support with shopping and domestic help, including services as Meals-On-Wheels or the presence of a person at mealtimes. To avoid that the person with early dementia might forget to eat, supervision during meals, verbal prompting and encouragement have been recommended. Feeding assistance and adaptations to meals (e.g., energy-dense foods) may provide further support in the eating process.

Interestingly, the interventions for treating malnutrition (or, better, preventing it (36)) in older persons with dementia are often based on social support, further enhancing the multifaceted dimension of the nutrition process. In this context, the assessment of the older person with nutritional status should become integral part of the clinical evaluation. There is indeed the necessity to train physicians at recognizing the signs of malnutrition and act against it, eventually integrating ad hoc professionals in the process. Meijers and colleagues (37) showed a significant reduction of malnutrition across clinical settings after regular activities of auditing and monitoring were implemented. Surely, the busy activities and possible limitations of specific settings may not facilitate the evaluation that, after all, is not even adequately explained at the schools of medicine (traditionally focused at teaching about diseases and drugs rather than preliminarily providing the basic knowledge to promote healthy aging!).

Importantly, the need of acting in a comprehensive, multidisciplinary, and coordinated way is the topic of the WHO document Integrated Care for Older PEople (ICOPE) [38], where a specific section is dedicated to malnutrition. It is here explained that nutritional supplements may surely play a role in recovering from severe deficiencies, although malnutrition requires a novel model of care for being tackled. The clinical condition typical of advanced age thus becomes a trigger for public health reorganization (39, 40, 41).

Beyond focusing on the effects of dementia on the individual's nutritional status, the interest of the scientific community is increasingly moving to the reciprocal perspective. That is, on the possibility of preventing or delaying the progression of dementia through nutritional interventions. In this regard, an international panel of experts recently convened that Souvenaid (a multinutrient product targeting synaptic dysfunction containing long-chain omega-3 fatty acids, uridine, choline, B vitamins, vitamin C, vitamin E, and selenium) should be considered as a therapeutic option in patients with prodromal AD or mild AD dementia (42). This advice was based on the results of randomized controlled trials of Souvenaid providing some evidence of efficacy in participants with early AD, but not among those at the advanced stages of the disease (43, 44, 45). These trials, although mostly failing to demonstrate any significance on the adopted cognitive primary outcomes, showed that the product was well tolerated and documented significantly less worsening in the active treatment groups. Specifically, in the LipiDiDiet study, 311 subjects with prodromal AD were recruited and randomized to a 24-month treatment period with an optional 12-month doubleblind extension (43). There was no statistically significant difference between the Souvenaid and control group for a neuropsychological composite score (i.e., the trial primary endpoint) or on the conversion to dementia. Nevertheless, participants in the active group exhibited less cognitive and functional worsening and less reduction in hippocampal volumes. Despite these promising findings, several issues remain to be addressed and seem to somehow limit the validity and widespread adoption of expert recommendations. Indeed, the clinical meaningfulness of the observed cognitive changes still needs to be clarified. In other words, it is not clear if the measured cognitive and functional benefits were actually responsible for a real clinical improvement. In addition, cost-efficacy analyses should be performed in order to explore the sustainability of the intervention for our healthcare systems. Finally, the “real world” transferability of the available evidence, obtained in highly selected populations of individuals with positive biomarkers, is questionable.

In conclusion, persons with early dementia do not substantially differ in needs and priorities from community-dwelling older persons. Under this perspective, dementia is only one of the many possible clinical conditions of old age. It is thus important to contextualize each case. This can be done by approaching the individual's nutritional status in a comprehensive way in order to develop person-tailored interventions nested in a multidisciplinary and integrated model of care. The most perfectly designed pharmacological intervention will always fail if the fundamental basis of healthy living (i.e., social network, physical activity, healthy diet) are weak.
